# MicroRNA-182-5p relieves murine allergic rhinitis via TLR4/NF-κB pathway

**DOI:** 10.1515/med-2020-0198

**Published:** 2020-12-24

**Authors:** Aichun Zhang, Yangzi Jin

**Affiliations:** Department of Otolaryngology, The First Affiliated Hospital of Zhejiang Chinese Medical University, 54 Youdian Road, Shangcheng, Hangzhou, Zhejiang 310000, People’s Republic of China

**Keywords:** allergic rhinitis, microRNA-182-5p, TLR4, NF-κB signaling pathway

## Abstract

Allergic rhinitis (AR) is one of the most common chronic diseases. This study examined whether microRNA (miR)-182-5p plays a role in AR by regulating toll-like receptor 4 (TLR4). First, data demonstrated that TLR4 was a target of miR-182-5p. Subsequently, AR mouse model was established to explore the role of miR-182-5p and TLR4 in AR *in vivo*. Initially, quantitative reverse transcription-PCR (qRT-PCR) analysis indicated that miR-182-5p was downregulated, while TLR4 expression was upregulated in AR mice. Then we found that miR-182-5p mimic reduced the frequency of sneezing and nose rubbing of the AR mice. In addition, miR-182-5p mimic significantly increased ovalbumin (OVA)-specific IgE and leukotriene C4 expression levels in nasal lavage fluid (NLF) and serum of AR mice. miR-182-5p mimic decreased the number of inflammatory cells in NLF of AR mice. It also reduced the levels of inflammatory factors in the serum of AR mice, such as interleukin (IL)-4, IL-5, IL-13, IL-17 and tumor necrosis factor (TNF)-α, while increasing the release of IFN-γ and IL-2. Finally, miR-182-5p mimic inhibited NF-κB signaling pathway activation in AR mice. However, all effects of miR-182-5p mimic on AR mice were reversed by TLR4-plasmid. In conclusion, miR-182-5p/TLR4 axis may represent a novel therapeutic target for AR.

## 1 Introduction

Allergic rhinitis (AR) is one of the most common chronic diseases. Its main feature is nasal mucositis, which is the most common non-infectious rhinitis driven by an immune response mediated by immunoglobulin E (IgE) [1]. AR induces upper respiratory tract inflammation, which is associated with release of mediators by several types of hypersensitive immune cells, including antigen-presenting cells, eosinophils, B cells and mast cells [2]. Several AR patients are usually accompanied by complications, such as chronic rhinosinusitis and asthma [3]. To a large extent, AR disease has affected social life and caused significant economic burden to families [1]. It has also reduced the quality of life of patients [1]. With the advancement of medical treatment, several therapeutic options have emerged for AR, including intranasal steroids, antihistamines, leukotriene receptor antagonists and immunotherapy [4,5]. However, the treatment options for 20% of AR patients remain suboptimal [6]. Therefore, a novel approach has to be developed for the effective treatment of AR.

At present, increasing evidence has shown that microRNAs (miRNAs) play crucial roles in the development of various diseases [7], such as cancer [8], atherosclerosis [9] and cardiovascular diseases [10]. miRNAs are a general term used for a class of small-molecule non-coding RNAs that are approximately 20 to 22 nucleotides in length, as opposed to mRNA-transcribed proteins. miRNAs do not encode proteins and inhibit the expression levels of multiple target genes by binding to their 3 prime-translated region (3′-UTR) [11,12,13]. miR-182-5p is a member of the miR-183 family. It has been reported that miR-182-5p participates in the development of various diseases, including cancer [14], heart disease [15] and acute lung injury [16]. Li et al. [14] demonstrated that miR-182-5p is an oncogene involved in the development of non-small cell lung cancer. Elena et al. [15] highlighted that miR-182-5p was highly expressed in the heart tissues of the HOS mouse model [16]. Zhu et al. reported that miR-182-5p suppressed the inflammatory response via the TLR4. However, to the best of our knowledge, the involvement of miR-182-5p in AR has not been previously examined.

TLR4 is a member of the TLR family of receptors. TLRs are pattern recognition receptors that participate in innate and acquired immune responses [17,18]. Currently, the most studied protein in the TLR family is the TLR4 protein, which is mainly distributed in the cell membrane and cytoplasm and plays an important role in immune cells [19]. A previous study indicated that TLR4 was overexpressed in AR and that low expression of this receptor played a protective role in the development of AR [20]. Increasing evidence has shown that TLR4 is a direct target gene of miR-182-5p.

Therefore, this study examined whether miR-182-5p played an important role in AR by regulating TLR4 expression. In this study, the role of miR-182-5p in AR and its potential interaction with the TLR4 were investigated.

## 2 Materials and methods

### 2.1 Establishment of ovalbumin (OVA) – induced AR model establishment

Six-week-old BALB/c mice were obtained from the Experimental Animal Center of Nanjing University. The mice were kept in a sterile environment at a temperature of 20 ± 1°C and a 12 h dark/light cycle. All mice received an excess of water and food. All animal experiments were performed according to a protocol approved by the Committee on Care and Use of Laboratory Animals of the First Affiliated Hospital of Zhejiang Chinese Medical University. The mice were sensitized for the first time on days 0, 7 and 14 by intraperitoneal injection of 200 μL saline containing 25 μg OVA and 2 mg aluminum hydroxide. One week after the last intraperitoneal injection, the mice were challenged intranasally daily and 3% OVA was diluted in 20 μL of saline for a second immunization. The mice of the control group were injected with saline without OVA and aluminum.

### 2.2 Intranasal administration

The total amount used for intranasal administration was usually 20 μL with *in vivo* transfection reagent (Entranster™-*in vivo*; Engreen Biosystem Co, Ltd) according to the procedure provided by the manufacturer. During the experiment, mimic control (5′-UUGUACUACACAAAAGUAGUC-3′; Guangzhou RiboBio Co., Ltd), miR-182-5p mimic (5′-UUUGGCAAUGGUAGAACUCACACCG-3′; Guangzhou RiboBio Co., Ltd), control-plasmid (cat no. sc-437275; Santa Cruz Biotechnology), TLR4 plasmid (cat no. sc-423419-ACT; Santa Cruz Biotechnology), miR-182-5p mimic + control-plasmid or miR-182-5p mimic + TLR4 plasmid were intranasally administered to mice 3 h before the OVA challenge on days 28–34. The mice of the AR group received 20 µL saline intranasally 3 h before every daily OVA challenge (once a day) on days 28–34.

### 2.3 Quantitative reverse transcription PCR (qRT-qPCR) assay

Total RNA was acquired using TRIzol (Invitrogen; Thermo Fisher Scientific, Inc.) according to the procedure provided by the manufacturer. Subsequently, RNA was reverse transcribed into complementary DNA (cDNA) using the reverse transcription kit (Vazyme) and cDNA was used for amplification. RT-qPCR was performed with the SYBR Green PCR kit (Vazyme). The amplification conditions were as follows: 95°C for 10 min, 35 cycles of 95°C for 15 s and 55°C for 40 s. GAPDH (for mRNA) or U6 (for miRNA) was used as an endogenous control. Primer sequences were as follows:

miR-182-5p forward, 5′-GTCGTATCCAGTGCGTGTCGTGGAGTC-3′;

reverse, 5′-GGCAATTGCACTGGATACGACAGTGTG-3′;

U6 forward, 5′-GCTTCGGCAGCACATATACTAAAAT-3′;

reverse, 5′-CGCTTCACGAATTTGCGTGTCAT-3′;

TLR4, forward 5′-CCTGACACCAGGAAGCTTGAA-3′;

reverse 5′-TCTGATCCATGCATTGGTAGGT-3′;

p65 forward 5ʹ-AGGCAAGGAATAATGCTGTCCTG-3ʹ;

reverse 5ʹ-ATCATTCTCTAGTGTCTGGTTGG-3ʹ;

GAPDH, forward 5′-CTTTGGTATCGTGGAAGGACTC-3′;

reverse 5′-GTAGAGGCAGGGATGATGTTCT-3′.

The 2^−ΔΔCq^ method [21] was used to quantify the relative gene expression. All samples were performed in three replicates and all experiments were repeated three times.

### 2.4 Western blot assay

The cells were lysed and total protein was obtained using RIPA buffer (Beyotime Institute of Biotechnology). A bicinchoninic acid assay kit (Pierce; Thermo Fisher Scientific, Inc.) was used to quantify the total protein. Equal amount of proteins were separated by 12% SDS–PAGE for 40 min and subsequently transferred to PVDF membranes (EMD Millipore). The membranes were blocked for 1.5 h at room temperature with 5% non-fat milk and incubated with primary antibodies including anti-TLR4 (cat no. ab13556; 1:1,000; Abcam), anti-p65 (cat no. ab32536; 1:1,000; Abcam) and anti-p-p65 (cat no. 194921; 1:1,000; Abcam) antibodies. The following day, the membranes were incubated with the secondary antibody (cat no. 7074; 1:2,000; Cell Signaling Technology, Inc.) for 2 h at room temperature. The protein bands were visualized by the enhanced chemiluminescence method (GE Healthcare Life Sciences). GAPDH (cat no. 181602; 1:1,000; Abcam) was used as a loading control for normalization of the protein levels.

### 2.5 ELISA

The levels of the following pro-inflammatory factors were measured: tumor necrosis factor-α (TNF-α; cat no. PT512; Beyotime Biotechnology), interleukin (IL)-2 (cat no. PI575; Beyotime Biotechnology), IL-4 (cat no. PI612; Beyotime Biotechnology), IL-5 (cat no. PI620; Beyotime Biotechnology), IL-13 (cat no. PI627; Beyotime Biotechnology), IL-17 (cat no. PI545; Beyotime Biotechnology), interferon-γ (IFN-γ; cat no. PI508; Beyotime Biotechnology), OVA-specific IgE (cat. no. 439807-1; BioLegend, Inc.) and leukotriene C4 (LTC4; cat. no. E-EL-M0753 km^−1^; Shanghai Zhenyu Chemical Technology Co., Ltd). The measurement was performed in mice of different groups by ELISA specific kits according to the manufacturer’s instructions. Each experiment was repeated at least three times.

### 2.6 Cell culture

HEK293T cells were obtained from the Shanghai library of the Chinese academy of sciences. HEK293T cells were cultured in DMEM medium (Invitrogen; Thermo Fisher Scientific, Inc.) supplemented with 10% FBS (Invitrogen; Thermo Fisher Scientific, Inc.) and 1% (v/v) penicillin–streptomycin (Gibco; Thermo Fisher Scientific, Inc.) in a humidified atmosphere containing 5% CO_2_ at 37°C.

### 2.7 Dual-luciferase reporter assay

The wild-type or mutant 3′-UTR of TLR4 was cloned into the pmiRGLO vector (Promega Corporation). The recombinant plasmids were acquired using the EndoFree Plasmid Maxi Kit (Vazyme). The cells were seeded in 24-well plates and co-transfected with miR-182-5p mimic or mimic control and the MUT or WT 3′-UTR sequences of TLR4 using Fugene transfection reagent (Promega Corporation) for 48 h. The *Renilla* luciferase pRL-TK vector was used as a control. Following transfection, the cells were incubated for 48 h and *Renilla* luciferase activity was tested using the dual-luciferase reporter assay (Promega Corporation) according to the manufacturer’s protocol. Firefly luciferase activity was normalized to *Renilla* luciferase activity.

### 2.8 Evaluation of nasal symptoms

The frequency of sneezing and nose rubbing of the mice was recorded 30 min after the last OVA challenge. The scores were calculated as follows: (i) slight rubbing of the nose several times or sneezing less than three times; (ii) repeated nose sneezing more than three times and less than 10 times; (iii) sustained rubbing of the nose to face or sneezing for 11 times as above. The animals that failed to score more than 4 points were excluded from further research.

### 2.9 Inflammation cell counting

The cells were re-suspended in 1 mL of 100 mM PBS and 1% BSA in nasal lavage fluid (NLF). The number of leukocytes was counted using a hemocytometer according to the manufacturer’s protocol. Subsequently, Wright’s–Giemsa staining (Beyotime Institute of Biotechnology) was performed at 37°C for 20 min and the number of eosinophils, neutrophils and lymphocytes was estimated with a light microscope at a magnification of ×200.

### 2.10 Statistical analysis

Every experiment was performed at least three times. All data were shown as mean ± SD. The significance of the differences between the groups was measured using the Student’s *t*-test or the one-way analysis of variance followed by a Tukey’s test. Statistical significance was set at *P* < 0.05. Data analyses were performed using GraphPad Prism 6.0.

## 3 Results

### 3.1 TLR4 is a target gene of miR-182-5p

**Figure 1:**
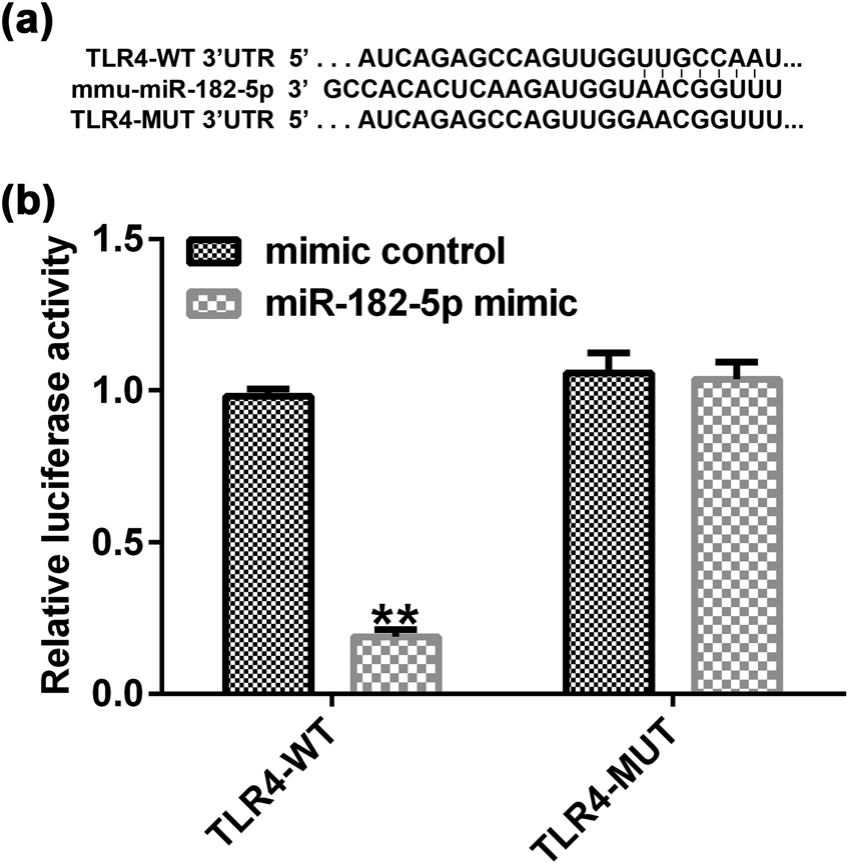
TLR4 is a target gene of miR-182-5p. (a) Prediction of miR-182-5p targeting TLR4 by the miRNA target gene database TargetScan. (b) The dual luciferase reporter gene assay was used to verify the association between miR-182-5p and TLR4 when 293T cells were transfected with miR-182-5p mimic and wild-type or mutant TLR4 3′-UTR reporter (*n* = 3). TLR4, toll-like receptor 4; miRNA, microRNA.

The underlying mechanism of TLR4 in AR was examined by the bioinformatic prediction algorithm TargetScan. The results indicated that TLR4 may be a target gene downstream of miR-182-5p (Figure 1a). Subsequently, the dual-luciferase activity assay was used to assess the association between these two targets. The 3′-UTR (wild-type or mutant) sequence of TLR4 was inserted into a pmiR luciferase reporter and 293T cells were co-transfected with miR-182-5p mimic or mimic control and TLR4-WT or TLR4-MUT. In addition, the results indicated that miR-182-5p mimic co-transfection with wild-type TLR4 3′-UTR reporter inhibited luciferase activity, whereas miR-182-5p mimic did not exert a significant effect on the reporter containing the mutant TLR4 (Figure 1b). Taken together, the data proved that TLR4 was the target gene of miR-182-5p.

### 3.2 Expression levels of miR-182-5p and TLR4 in the nasal mucosa of AR mice

**Figure 2:**
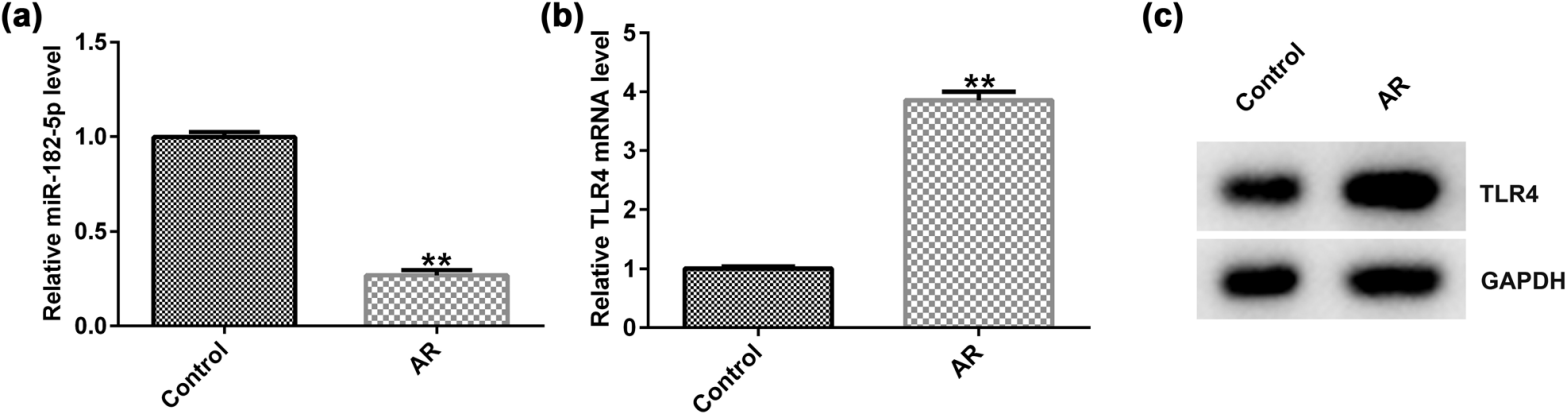
miR-182-5p expression is downregulated and TLR4 is upregulated in AR mice. (a) qRT-PCR analysis was used to detect miR-182-5p expression in the untreated and AR groups (*n* = 3). (b) qRT-PCR analysis was used to detect TLR4 expression in the untreated and AR groups (*n* = 3). (c) Western blot analysis was used to detect TLR4 protein expression. TLR4, toll-like receptor 4; qRT-PCR, quantitative reverse transcription-PCR.

To explore the role of miR-182-5p and TLR4 in AR, a mouse model of AR was established. qRT-PCR and western blot assays were performed to detect miR-182-5p and TLR4 expression. qRT-PCR analysis indicated that miR-182-5p expression was downregulated (Figure 2a), whereas TLR4 expression was upregulated (Figure 2b) in the nasal mucosa of AR mice compared with the corresponding expression noted in the untreated group. Western blot analysis indicated that TLR4 protein expression was increased in AR mice (Figure 2c).

### 3.3 Effects of miR-182-5p mimic and TLR4-plasmid cell transfection on miR-182-5p and TLR4 expression in the nasal mucosa of AR mice

**Figure 3:**
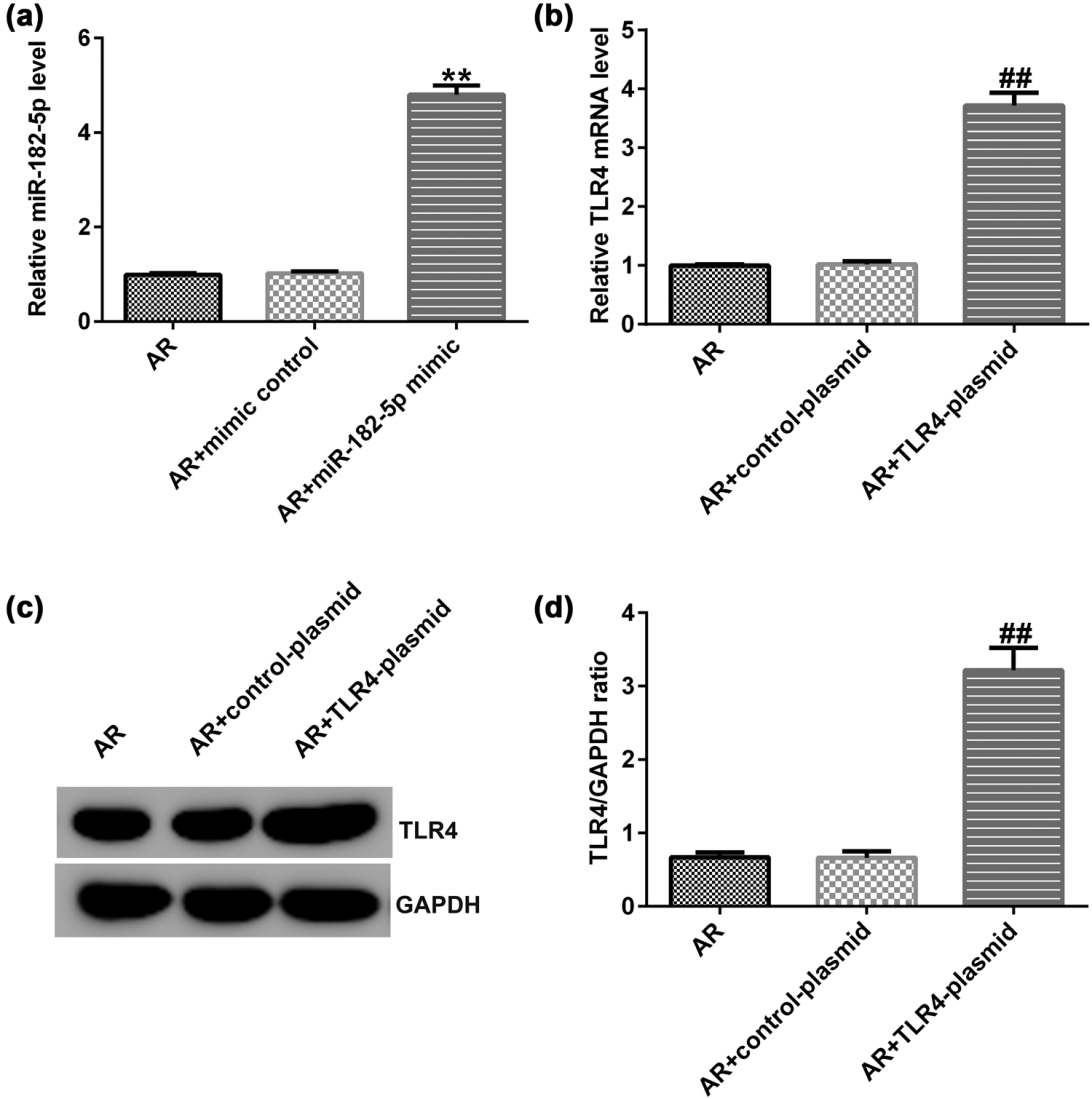
miR-182-5p mimic and TLR4 plasmid increase miR-182-5p and TLR4 expression in AR mice. (a) qRT-PCR analysis was used to detect miR-182-5p expression in the AR group; AR + mimic control group and AR + miR-182-5p mimic group (*n* = 3). (b) qRT-PCR analysis was used to detect TLR4 expression in the AR group; AR + control-plasmid group and AR + TLR4 plasmid group (*n* = 3). (c) Western blot analysis for the detection of TLR4 protein levels. (d) TLR4/GAPDH ratio was calculated and presented. TLR4, toll-like receptor 4; qRT-PCR, quantitative reverse transcription-PCR; AR, allergic rhinitis.

Following the establishment of the AR mouse model, different intranasal administration was performed on AR mice. Initially, mimic control or miR-182-5p mimic sequences were administered into the nostril daily for 7 days, 3 h before OVA challenge on days 28–34. qRT-PCR analysis indicated that miR-182-5p mimic significantly increased miR-182-5p expression in AR mice (Figure 3a). qRT-PCR assay and western blot analyses indicated that the transfection of the TLR4 plasmid to the cells significantly increased TLR4 expression in AR mice (Figure 3b–d). Therefore, intranasal administration of miR-182-5p mimic and TLR4 plasmid resulted in the increase of their expression levels in AR mice.

### 3.4 TLR4 reverses the mitigating effect of miR-182-5p on AR mice

**Figure 4:**
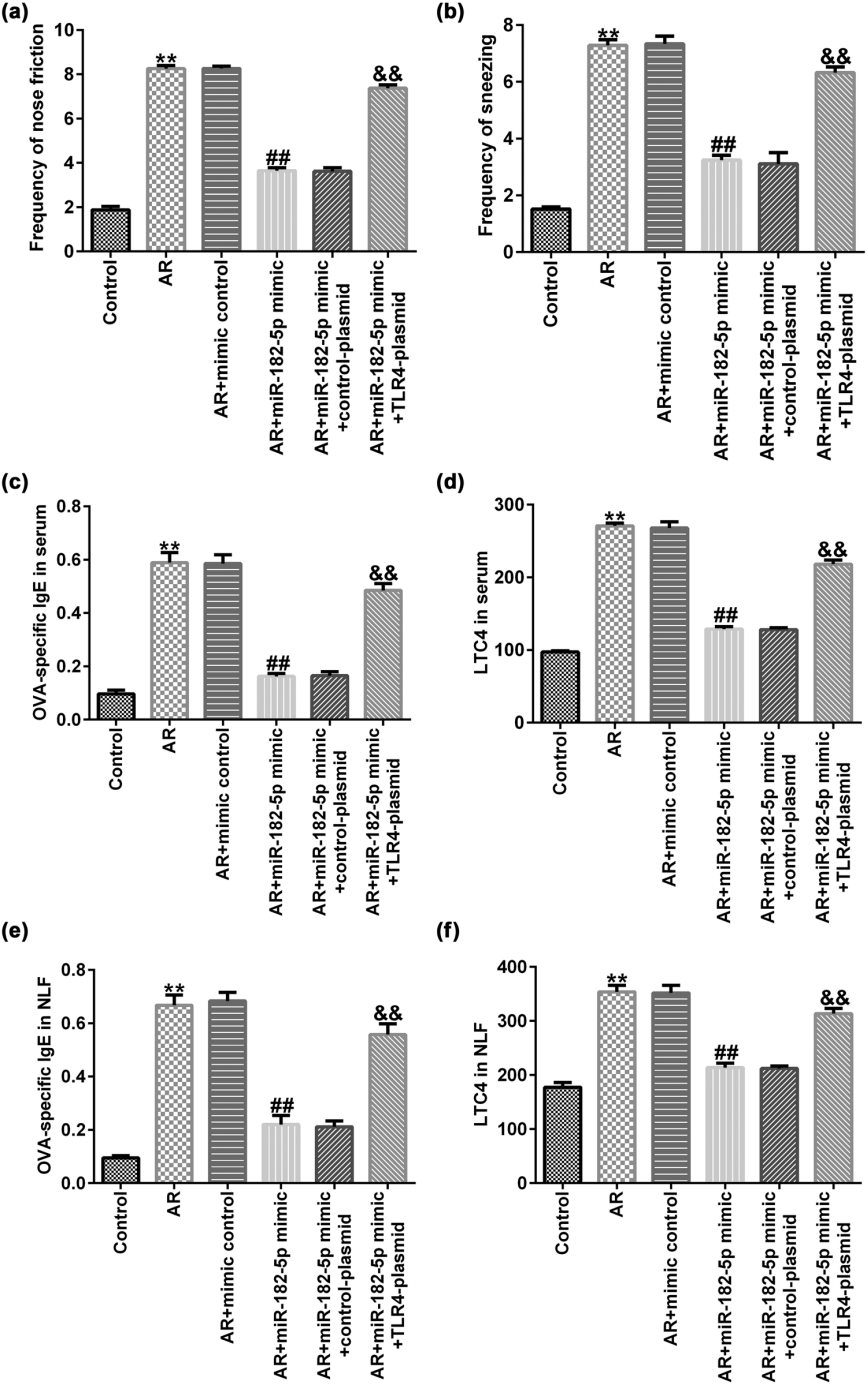
TLR4 reverses the mitigating effect of miR-182-5p on AR mice (*n* = 3). (a) The frequency of nose rubbing was recorded within 10 min after the last OVA attack on day 34 and the symptom score was calculated as described in the materials and methods section. (b) The frequency of sneezing was recorded. (c and e) The expression levels of OVA-specific IgE were detected using an ELISA kit. (d and f) The expression levels of LTC4 were detected using an ELISA kit. TLR4, toll-like receptor 4; AR, allergic rhinitis; OVA, ovalbumin, IgE, immunoglobulin E, LTC4, leukotriene C4.

To explore the effects of miR-182-5p and TLR4 on the allergic response of AR mice, the frequency of sneezing and nose rubbing was examined. Moreover, the expression levels of allergic inflammatory cytokines and OVA-specific IgE were assessed in AR mice. Following the last sensitization on day 34, the symptoms of nasal allergies were counted. The frequency of sneezing and nose rubbing of the mice was significantly increased in the AR group compared with that of the untreated group; miR-182-5p mimic reduced the frequency of sneezing (Figure 4a) and nose rubbing (Figure 4b) of the mice compared with that of the AR group. These changes were reversed by transfection of the TLR4 plasmid. Furthermore, the expression levels of OVA-specific IgE and leukotriene C4 (LTC4) in the serum and the nasal lavage fluid of the AR group were significantly increased compared with those of the control group; miR-182-5p mimic reduced the expression levels of OVA-specific IgE (Figure 4c and e) and LTC4 (Figure 4d and f) compared with those of the AR group. These changes were reversed by transfection of the TLR4 plasmid to the cells.

### 3.5 miR-182-5p affects the number of inflammatory cells in AR mice

**Figure 5:**
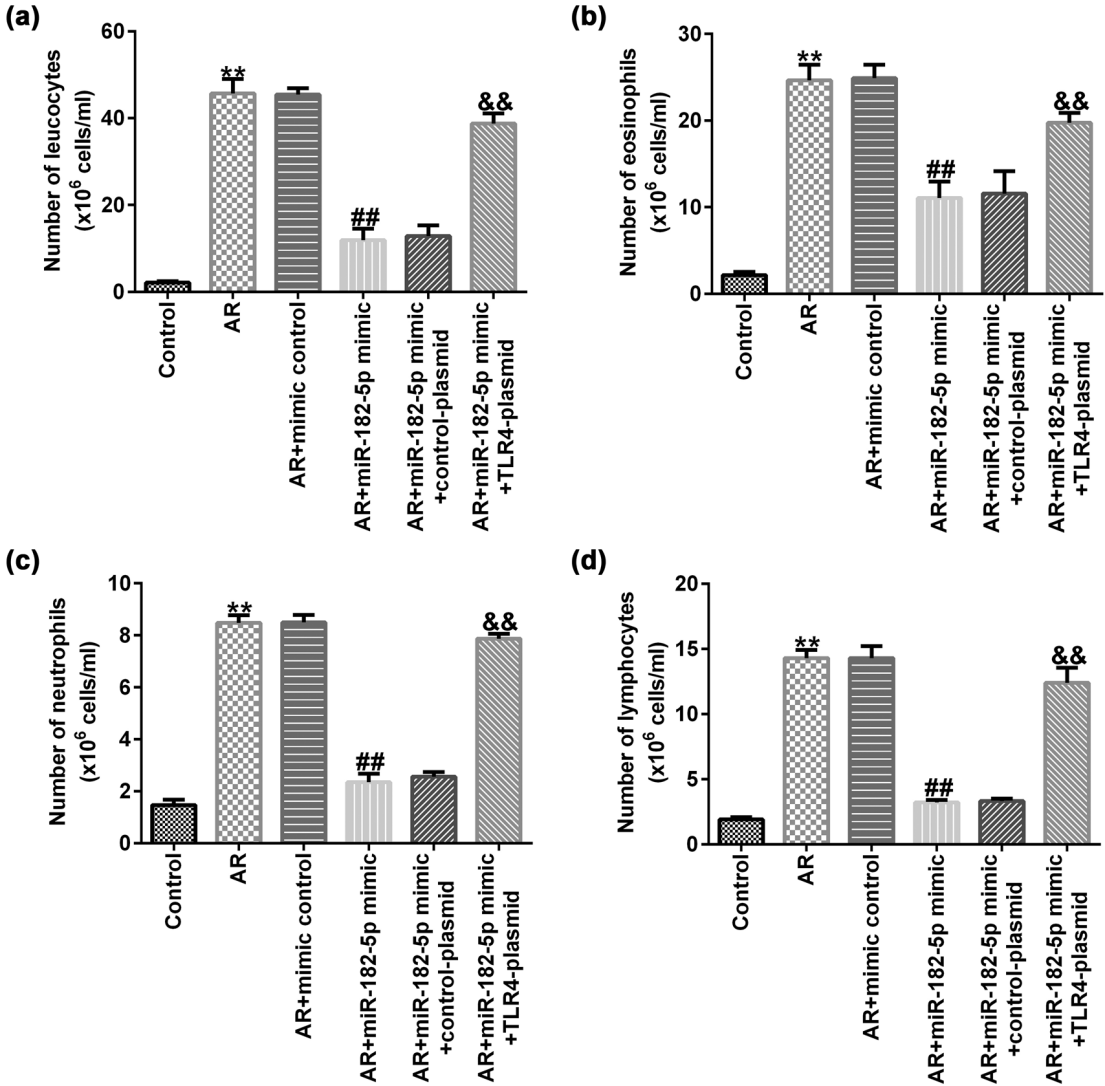
TLR4 reverses the inhibitory effect of miR-182-5p on inflammatory cells in AR mice (*n* = 3). (a) The number of leukocytes was counted using a hemocytometer. The number of eosinophils (b), neutrophils (c) and lymphocytes (d) was counted with the Wright’s–Giemsa staining. TLR4, toll-like receptor 4; AR, allergic rhinitis.

The effects of miR-182-5p on the number of inflammatory cells were investigated. The number of white blood cells, eosinophils, neutrophils and lymphocytes was increased in the nasal lavage fluid (NLF) of the AR mice compared with that in the untreated group. miR-182-5p mimic significantly decreased the number of leukocytes (Figure 5a), eosinophils (Figure 5b), neutrophils (Figure 5c) and lymphocytes (Figure 5d) in the NLF of AR mice compared with that in the AR group. These changes were reversed by transfection of the TLR4 plasmid to the cells.

### 3.6 miR-182-5p affects inflammatory cytokine release in AR mice

**Figure 6:**
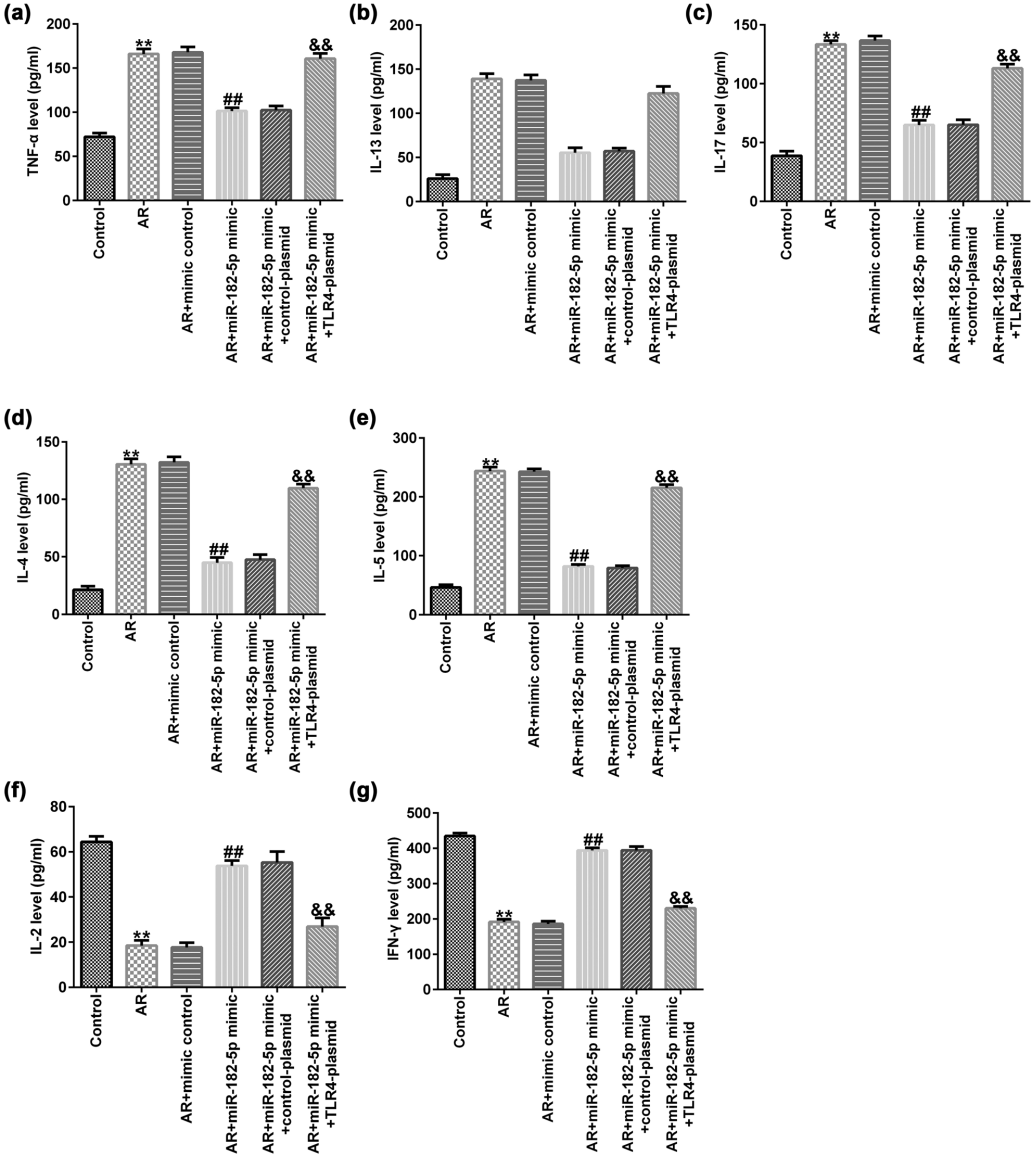
TLR4 reversed the effect of miR-182-5p on the expression of inflammatory cytokines in AR mice (*n* = 3). (a–g) The expression levels of TNF-α, IL-13, IL-17, IL-4, IL-5, IL-2 and IFN-γ in the serum samples from different groups. TLR4, toll-like receptor 4; AR, allergic rhinitis; TNF-α, tumor necrosis factor-α; IL13, interleukin 13; IL-17, interleukin 17; IL-4, interleukin 4; IL-5, interleukin 5; IL-2, interleukin 2.

The expression levels of the inflammatory factors including TNF-α, IL-13, IL-17, IL-4 and IL-5 were significantly increased in the serum of the AR mice compared with those in the control group. In addition, the release of IL-2 and IFN-γ was significantly reduced compared with that in the control group. miR-182-5p mimic significantly decreased the expression levels of certain inflammatory factors, such as TNF-α (Figure 6a), IL-13 (Figure 6b), IL-17 (Figure 6c), IL-4 (Figure 6d) and IL-5 (Figure 6e) compared with those of the AR group. Concomitantly, it increased IL-2 (Figure 6f) and IFN-γ (Figure 6g) release. These changes were significantly reversed by transfection of the TLR4 plasmid to the cells.

### 3.7 miR-182-5p suppresses the activation of the NF-κB signaling pathway

**Figure 7:**
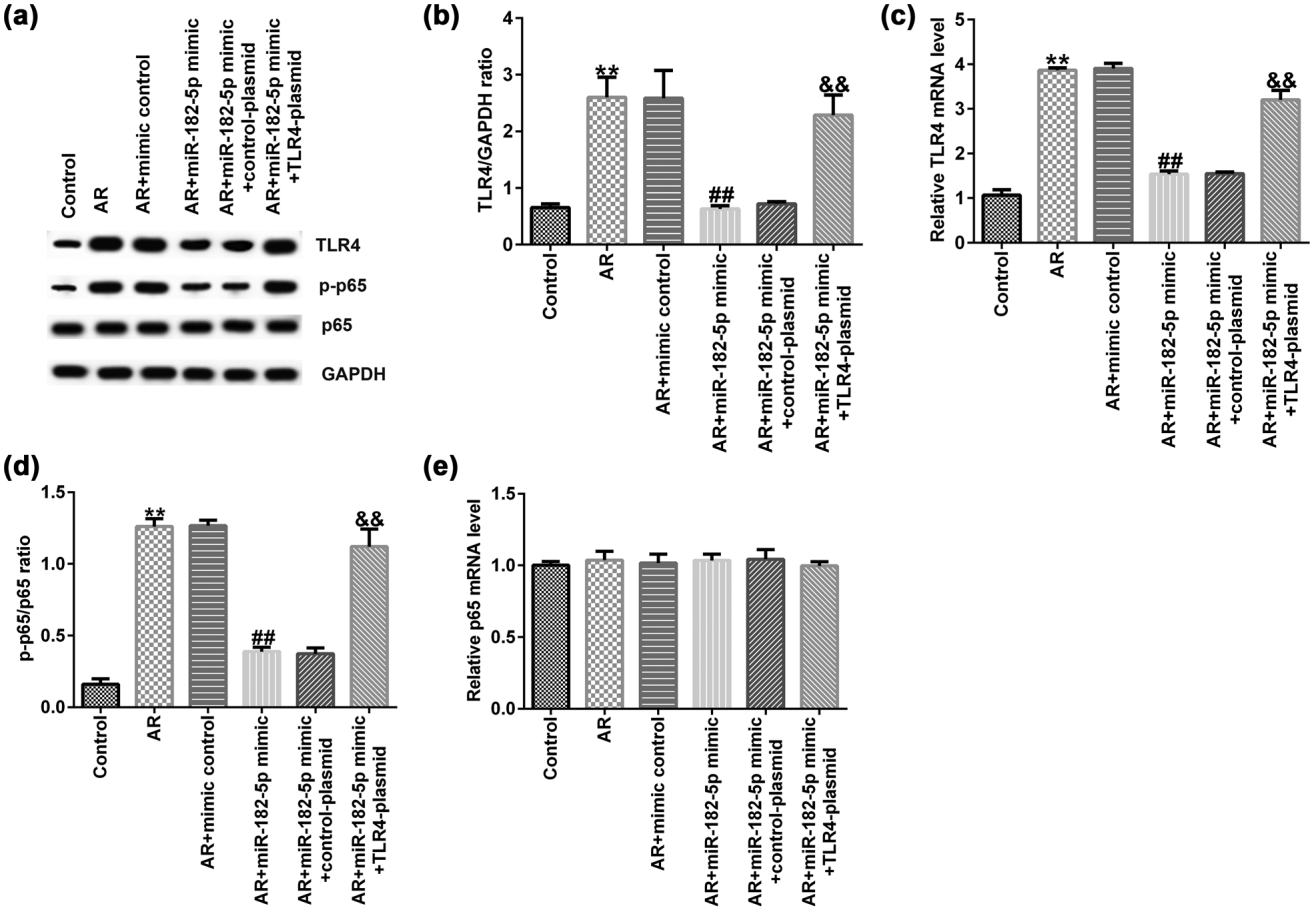
TLR4 reversed the inhibitory effect of miR-182-5p on the NF-κB signaling pathway. (a) Western blot analysis was used to determine TLR4, p65 and p-p65 protein expression. (b) TLR4/GAPDH ratio was calculated and presented (*n* = 3). (c) RT-qPCR analysis was used to assess TLR4 mRNA expression (*n* = 3). (d) The ratio of p-p65/p65 was calculated (*n* = 3). (e) qRT-PCR analysis was used to detect p65 mRNA levels (*n* = 3). TLR4, toll-like receptor 4; qRT-PCR, quantitative reverse transcription-PCR.

Finally, the interaction of miR-182-5p with the NF-κB signaling pathway was examined in AR mice. The data indicated that the protein expression levels of TLR4 and p-p65 were significantly increased in the nasal mucosa of AR mice compared with those of the untreated group; the mRNA expression levels of TLR4 were increased and the p-p65/p65 ratio was significantly increased in the AR mice compared with the corresponding levels in the control group. miR-182-5p mimic significantly decreased the protein expression levels of TLR4 (Figure 7a and b) and p-p65 (Figure 7a) and reduced the mRNA expression levels of TLR4 (Figure 7c) and the p-p65/p65 ratio (Figure 7d) compared with those in the AR group. These effects were significantly reversed by transfection of the TLR4 plasmid to the cells. In addition, the protein and mRNA expression levels of p65 did not reveal significant differences among different groups (Figure 7e).

## 4 Discussion

In the past years, miRNAs have been reported to be involved in the occurrence and development of a variety of diseases [22,23]. A previous study has shown that miRNAs can bind to the 3′-UTR of the target gene mRNAs and degrade the expression of homologous target genes at the post-transcriptional level [24]. Since miRNAs have higher stability than several mRNA groups or proteins, certain investigators proposed that miRNA stability may regulate gene expression in several diseases including AR [23,25]. AR is one of the most common inflammatory diseases that mainly occur in specific seasons (spring and autumn). The average incidence range of AR was estimated as 10–20% in 2013 (25–26). These percentages are increasing every year [26,27]. In recent years, the incidence of AR has increased and this disease has become a serious health problem that requires immediate treatment [28].

Liu et al. [29] highlighted that miR-487b overexpression played an inhibitory role in AR by targeting IL-33. Wang et al. [30] demonstrated that miR-202-5p participated in the development of AR by targeting MATN2. Xiao et al. demonstrated that miR-302e suppressed allergic inflammation by inhibiting the NF-κB signaling pathway. However, the role of miR-182-5p in AR has not been previously reported. This study confirmed that miR-182-5p played an important role in AR mice.

TLR4 is an innate and adaptive immune cell receptor [31] that plays a vital role in the inflammatory response [32]. Yang et al. [31] showed that TLR4 is a target of miR-760. Previous studies have indicated that TLR4 is a target gene of miR-182-5p [33,34]. In this study, similar findings were reported and TLR4 was identified as the target gene of miR-182-5p in AR. It has been reported that TLR4 can induce a pre-inflammatory immune response by recognizing pathogens and endogenous ligands [35]. However, abnormal expression of TLR4 is also involved in the development of various diseases [35]. Previous studies have shown that TLR4 expression is upregulated in the nasal mucosa of AR patients. Increasing evidence has shown that TLR4 participates in the development of AR [36,37].

In this study, miR-182-5p expression was downregulated in AR mice. However, TLR4 expression was upregulated. This result was consistent with the result of a previous study [38]. Subsequently, the effects of miR-182-5p and TLR4 were examined on the frequency of sneezing and nose rubbing of AR mice. miR-182-5p mimic significantly reduced the frequency of sneezing and nose rubbing in AR mice. This reduction was significantly reversed by transfection of the TLR4 plasmid to the cells. During the pathogenesis of AR, allergens trigger a Th2-based immune response, which produces antigen-specific IgE [39]. Repeated exposure to the same allergen activates IgE-bound mast cells, releasing inflammatory mediators including histamine and leukotriene C4 [40]. Yuan et al. [41] demonstrated that the expression levels of OVA-specific IgE and leukotriene C4 (LTC4) were upregulated in AR. This study confirmed this result. In addition, AR is an inflammatory disease, accompanied by inflammatory cell infiltration including basophilic mast cells and eosinophils [24]. In AR mice, the number of inflammatory cells is upregulated leading to their flow into the nasal mucosa and to the production of proinflammatory cytokines. In this study, miR-182-5p mimic significantly reduced the levels of the inflammatory factors IL-4, IL-5, IL-13, IL-17 and TNF-α in the serum of AR mice, while it increased the release of IFN-γ and IL-2, which was reversed following treatment with the TLR4 plasmid. Finally, this study demonstrated that the miR-182-5p/TLR4 axis was associated with the NF-κB signaling pathway. Previous studies indicated that activation of the TLR4/NF-κB pathway was critical for the pathogenesis of multiple lung diseases, such as ALI [42,43]. Evidence shows that miRNA can bind to the 3′-UTR of target gene mRNA and degrade the expression of the homologous target gene at the post-transcriptional level [24]. Consistent with previous studies, our data have indicated that TLR4 is a target gene of miR-182-5p [33,34]. Therefore, we believe that miR-182-5p can negatively regulate the expression of TLR4 by binding to the 3′-UTR of TLR4. As expected, miR-182-5p mimic significantly reduced TLR4 expression in AR mice, and this inhibition was reversed by TLR4-plasmid. Moreover, the findings indicated that miR-182-5p mimic inhibited NF-κB signaling pathway activation in AR mice. Taken together, the data revealed that the miR-182-5p/TLR4 axis was a promising therapeutic strategy that can be used for the treatment of AR.

In conclusion, miR-182-5p inhibited the activation of the NF-κB signaling pathway by targeting TLR4 and thereby played a protective role in AR.
